# Decrease in Skin Prion-Seeding Activity of Prion-Infected Mice Treated with a Compound Against Human and Animal Prions: a First Possible Biomarker for Prion Therapeutics

**DOI:** 10.1007/s12035-021-02418-6

**Published:** 2021-05-13

**Authors:** Mingxuan Ding, Kenta Teruya, Weiguanliu Zhang, Hae Weon Lee, Jue Yuan, Ayumi Oguma, Aaron Foutz, Manuel V. Camacho, Marcus Mitchell, Justin J. Greenlee, Qingzhong Kong, Katsumi Doh-ura, Li Cui, Wen-Quan Zou

**Affiliations:** 1 Department of Neurology, The First Hospital of Jilin University, Changchun, 130021 Jilin Province China; 2 Departments of Pathology and Neurology, Case Western Reserve University School of Medicine, Cleveland, OH 44106 USA; 3 Department of Neurochemistry, Tohoku University Graduate School of Medicine, 2-1 Seiryo-machi, Aoba-ku, Sendai, Miyagi 980-8575 Japan; 4 Virus and Prion Research Unit, Agricultural Research Service, National Animal Disease Center, USDA, 1920 Dayton Avenue, Ames, IA 50010 USA; 5 National Prion Disease Pathology Surveillance Center, Case Western Reserve University School of Medicine, Cleveland, OH 44106 USA

**Keywords:** Prions, Prion diseases, Cellulose ethers, Real-time quaking-induced conversion (RT-QuIC), Serial protein misfolding cyclic amplification (sPMCA), TC-5RW

## Abstract

Previous studies have revealed that the infectious scrapie isoform of prion protein (PrP^Sc^) harbored in the skin tissue of patients or animals with prion diseases can be amplified and detected through the serial protein misfolding cyclic amplification (sPMCA) or real-time quaking-induced conversion (RT-QuIC) assays. These findings suggest that skin PrP^Sc^-seeding activity may serve as a biomarker for the diagnosis of prion diseases; however, its utility as a biomarker for prion therapeutics remains largely unknown. Cellulose ethers (CEs, such as TC-5RW), widely used as food and pharmaceutical additives, have recently been shown to prolong the lifespan of prion-infected mice and hamsters. Here we report that in transgenic (Tg) mice expressing hamster cellular prion protein (PrP^C^) infected with the 263K prion, the prion-seeding activity becomes undetectable in the skin tissues of TC-5RW-treated Tg mice by both sPMCA and RT-QuIC assays, whereas such prion-seeding activity is readily detectable in the skin of untreated mice. Notably, TC-5RW exhibits an inhibitory effect on the in vitro amplification of PrP^Sc^ in both skin and brain tissues by sPMCA and RT-QuIC. Moreover, we reveal that TC-5RW is able to directly decrease protease-resistant PrP^Sc^ and inhibit the seeding activity of PrP^Sc^ from chronic wasting disease and various human prion diseases. Our results suggest that the level of prion-seeding activity in the skin may serve as a useful biomarker for assessing the therapeutic efficacy of compounds in a clinical trial of prion diseases and that TC-5RW may have the potential for the prevention/treatment of human prion diseases.

**Supplementary Information:**

The online version contains supplementary material available at 10.1007/s12035-021-02418-6.

## Introduction

Prion diseases or transmissible spongiform encephalopathies are a group of neurodegenerative diseases affecting the central nervous system of humans and animals, including Creutzfeldt-Jakob disease (CJD), kuru, fatal familial insomnia (FFI), Gerstmann-Sträussler-Scheinker (GSS) syndrome, and variably protease-sensitive prionopathy (VPSPr) in humans, and scrapie in sheep and goats, bovine spongiform encephalopathy (BSE), and chronic wasting disease (CWD) in elk and deer [1]. They have a long incubation period and a 100% fatality rate. All these diseases have detectable deposition in the brain of abnormal infectious misfolded prion protein (PrP^Sc^), a molecular hallmark of prion diseases, which is derived from its normal cellular prion protein (PrP^C^) through a structural transition [2].

Our previous study revealed that autopsy skin tissues from sporadic CJD cadavers harbor PrP^Sc^ that exhibited seeding activity and infectivity [3] that has recently been confirmed not only with autopsy skin samples from CJD cadavers diagnosed neuropathologically but also biopsy skin samples from living CJD patients [4]. Moreover, in animal models including 263 K scrapie prion-infected hamsters and sporadic CJD (sCJD) prion-infected humanized transgenic (Tg) mice expressing human wild-type PrP, we further observed that skin PrP^Sc^ was detectable by real-time quaking-induced conversion (RT-QuIC) and serial protein misfolding cyclic amplification (sPMCA) assays long before clinical signs and brain lesions manifested [5]. These observations provide the proof-of-concept that skin PrP^Sc^ may be a biomarker for early preclinical diagnosis of prion diseases. However, it is unclear whether the level of prion-seeding activity in the skin can serve as a biomarker for assessing the efficacy of anti-prion therapeutics.

Cellulose ethers (CEs), a family of non-digestible, non-ionic, and water-soluble polysaccharide derivatives, are widely used as additives in food, pharmaceutical tablets, and personal care products [6, 7]. Doh-ura and co-workers first discovered that CEs including TC-5RW have a significant protective effect on prion infection of animals that were given before and after inoculation [8]. Other teams have also confirmed that this compound can prolong the survival of infected rodents expressing elk or deer PrP infected with different CWD prions [9, 10]. Moreover, a liposomal formulation of CEs was found successfully to lower the effective dose of CE in prion-infected cells [11].

In the present study, we investigate prion-seeding activity in skin samples from prion-infected Tg mice with or without TC-5RW treatment via RT-QuIC and sPMCA assays. We find that the prion-seeding activity in the skin of TC-5RW-treated mice becomes undetectable but remains detectable in vehicle-treated control mice. Moreover, we reveal that TC-5RW inhibits the amplification of hamster PrP^Sc^ from skin and brain tissues in RT-QuIC and sPMCA experiments. Additionally, our RT-QuIC assay reveals that TC-5RW is able to inhibit the seeding activity of PrP^Sc^ from CWD and various human prion diseases as well. Finally, in vitro direct incubation of TC-5RW with brain homogenates from hamster, elk, deer, or humans infected by various prions is found to decrease the levels of protease-resistant PrP^Sc^ (PrP^res^).

## Materials and Methods

### Reagents and Antibodies

TC-5RW was kindly provided by Shin-Etsu Chemical Co., Ltd. (Tokyo, Japan). Proteinase K (PK) was purchased from Sigma ALDRICH Co. (St. Louis, MO, USA). Protease inhibition cocktail tablets were purchased from Roche Diagnostics (Indianapolis, IN, USA). Reagents for enhanced chemiluminescence (ECL Plus) were from Thermo Scientific (Rockford, IL, USA). Anti-PrP mouse monoclonal antibody 3F4 [12, 13] and sheep anti-mouse IgG conjugated with horseradish peroxidase as a secondary antibody were purchased from Sigma Aldrich [14].

### Animal Study

The animal experiment was performed following a protocol reviewed and approved by the Institutional Animal Care and Use Committee of Tohoku University (approval number 2016MdA-139). Thirteen hemizygous Tg7 mice at 6 to 10 weeks old obtained by a cross between Tg7 [15] and PrP-null mice [16] were inoculated intracerebrally with 20 μL of 1% (w/v) brain homogenate obtained from a terminally ill 263 K prion-infected hamster. On the day of inoculation, 8 mice were also injected with a single intraperitoneal injection of 1 mL of 50 mg/mL TC-5RW in saline, and the other 5 mice were inoculated with 1 mL of saline as vehicle-treated controls. Mice were monitored daily from inoculation until the terminal stage, at which time the mice exhibited akinesia (with a lack of grooming behavior, coordination, and parachute reaction) or exhibited a rigid tail, an arched back, and weight loss of approximately 10% within 1 week [17]. The mice were sacrificed at the terminal stage. The time from inoculation to death was defined as the survival time. Skin samples from the back near the tail and brain samples were taken immediately after sacrifice as described previously [5]. The scissors and tweezers used for skin collection were handled with great care to avoid contamination from the brain to the skin or between mice. The skin samples were collected first before opening the skull for collecting brain tissues and all devices were decontaminated after each use. The skin tissues were frozen on dry ice and stored in a − 80 °C freezer for future use. Some of the brain samples were similarly frozen and kept in a freezer, while some were formalin fixed and paraffin embedded as described previously [18].

### Paraffin-Embedded Tissue Blotting and Hematoxylin/Eosin Staining

Paraffin-embedded tissue (PET) blot analysis was performed for detection of PrP^Sc^ deposition in the brain as described previously [18]. In brief, 6-μm paraffin-embedded brain sections were cut and collected onto nitrocellulose membranes and then dried overnight at 60 °C. Membranes were dewaxed in xylene, followed by step-wise rehydration. After wetting with Tris-buffered saline-tween 20 (TBST) (10 mM Tris–HCl, pH 7.8, 100 mM NaCl, and 0.05% Tween-20), sections were digested with 250 μg/mL PK in a buffer (10 mM Tris–HCl, pH 7.8, 100 mM NaCl, and 0.1% Brij35) overnight at 55 °C. After washing with TBST, sections were treated with 3 M guanidine isothiocyanate for 30 min. After washing out guanidine using TBST, immunodetection was performed with an anti-PrP-C antibody, which recognizes residues 214–228 of mouse/hamster PrP (1:1500, Immuno-Biological Laboratories Co., Ltd., Gunma, Japan). The 3-μm paraffin-embedded brain sections serially adjacent to those used for PET blot analysis were stained with hematoxylin and eosin (H&E). Assessment of pathological changes including spongiform degeneration was conducted under a light microscope.

### Preparation of Brain and Skin Samples

Skin samples (~ 50 mg each in weight, ~ 5 mm × 5 mm each in size) were taken with epidermis, dermis, and hypodermis three layers as described previously [5]. They were washed three times in 1 × Tris-buffered saline (TBS) to remove possible blood contamination and cut into small pieces in dishes. The 5% (w/v) skin homogenates were prepared in skin lysis buffer containing 10 mM Tris–HCl, 133 mM NaCl, 2 mM CaCl2, and 0.25% collagenase A (Roche), pH 7.4, and incubated in a shaker at 37 °C for 4 h. Mouse brain samples at 5% (w/v), hamster, and human autopsy brain samples at 10% (w/v) were homogenized in lysis buffer containing 125 mM NaCl, 12.5 mM EDTA, 12.5 mM Tris–HCl, 0.5% sodium deoxycholate, 0.5% NP-40, and pH 7.4, with a Mini-Beadbeater (BioSpec, Laboratory Supply Network, Inc., Atkinson, NH) shaking (1 min)-incubating on ice (2 min) cycle for 3 cycles. Frozen brain tissues from two white-tailed deer and a reindeer with CWD [19–21] were prepared for 10% brain homogenate in 2% Sarkosyl solution.

### RT-QuIC Assay

RT-QuIC assays of skin from Tg7 mice and brain samples from 263 K-infected hamster, patients with different prion diseases, and CWD deer were performed as previously described [5], with minor modification. In brief, RT-QuIC reaction mix was composed of 1 × phosphate buffer pH 7.4, 0.17 M NaCl, 0.1 mg/mL homemade recombinant truncated Syrian golden hamster PrP90-231 [22], 10 μM Thioflavin T (ThT), 1 mM EDTA, and 0.001% SDS. Each well of a 96-well plate (Nunc) was loaded with 96 μL of reaction mix and seeded with 2 μL of Tg7 mouse skin homogenate at a final concentration of 10^−3^ or deer or human brain homogenate at a final concentration of 2 × 10^−7^. Seeds were spun at 5000 × *g* for 2 min at 4 °C prior to loading. To investigate the inhibitory effect of TC-5RW on prion-seeding activity in RT-QuIC, 2 μL of differing concentrations of TC-5RW was added to reaction wells before loading seeds. The plates were sealed with a plate sealer film (Nalgene Nunc International) and incubated at 55 °C for skin samples and 42 °C for brain samples in a BMG FLUOstar Omega plate reader with cycles of 1 min shaking (700 rpm double orbital) and 1-min rest throughout the indicated incubation time. ThT fluorescence intensity (450 ± 10-nm excitation and 480 ± 10-nm emissions; bottom read) was measured every 45 min. All samples were run in quadruplicate. The average fluorescence of each sample was determined by taking the average of all four replicates regardless of whether their ThT values were above the threshold described below. Samples with at least 2 out of 4 replicate wells above the determined threshold were considered positive. A ThT fluorescence threshold for a reaction to define the positive and negative cases was determined based on the mean ThT value of all negative control samples at 60 h, plus 3 standard deviations as previously described [5]. For comparison, the average ThT fluorescence was normalized as percentages with the highest fluorescence in each plate. The differences in prion-seeding activity may result in variable lag phase or lag time of prion aggregation, the time point when the ThT fluorescence of protein aggregates RT-QuIC starts to continuously increase [10]. So, the lag time of the RT-QuIC reaction was also used to compare prion-seeding activity among different prion diseases in our study.

### Serial PMCA Analysis

Serial PMCA (sPMCA) assay as well as the preparation of PrP^Sc^ seeds and PrP^C^ substrates were conducted as previously described [5, 23, 24] with minor modification. Briefly, to make the sPMCA PrP^C^ substrate, normal hamster or humanized Tg mouse brain tissues expressing human wild-type PrP (Tg40h) [13] were carefully dissected to avoid blood contamination as much as possible. The normal hamster or humanized Tg mouse brain tissues were homogenized (10% w/v) in sPMCA conversion buffer containing 150 mM NaCl, 1% Triton X-100, 8 mM EDTA, pH 7.4, and the complete protease inhibitor mixture cocktail (Roche) in PBS, followed by centrifugation at 1000* g* at 4 °C for 10 min to collect the supernatant (S1) fraction. Finally, the supernatant was mixed with 5 mg/mL heparin at 50:1. The substrates and seeds were kept at − 80 °C until use. Each skin PrP^Sc^ seed was diluted in the substrate at the ratios 1:12.5 (8 μL seed in 100 μL mix) into 200 μL PCR tubes with 1 PTFE beads (diameter 3/32ʺ) (Teflon, APT, RI) while 263 K-infected hamster brain homogenate seeds or sCJDMM1- or sCJDMM2-infected human brain homogenate seeds were diluted at the ratios 1:100 (1 μL seed in 100 μL mix) as positive controls. To detect the inhibitory effect of TC-5RW of seed activity in sPMCA, 2 μL different concentration of TC-5RW was added to the 100 μL system. A 20 μL of each mixture was taken out and kept at – 20 °C as a non-PMCA control. The remaining mixtures were subjected to sPMCA. Each cycle comprises a 20-s elapse time of sonication at amplitude 85 (250 W; Misonix S4000 sonicator) followed by an incubation period of 29 min 40 s at 37 °C and each round of sPMCA consisted of 80 cycles. For the sPMCA, 15-μL sample, each was aliquoted from the last cycle and placed into 65-μL fresh normal brain substrates for the next round of amplification.

### Incubation of TC-5RW with Brain Homogenates

Ten percent (w/v) brain homogenates in lysis buffer from patients with different sCJD subtypes or 263 K-infected hamsters were incubated with TC-5RW at final concentrations ranging from 0 ~ 30 μg/mL for 37 °C, 400 rpm, or kept at – 20 °C as designated hours. The samples were treated with PK at 100 μg/mL, 37 °C for an hour, followed by protease inhibition cocktail and boiled in the SDS sample buffer prior to Western blot analysis probing with the 3F4 antibody as shown below.

### Two-Dimensional Western Blotting

Two-dimensional (2D) Western blotting of PrP was performed as previously described [14]. In brief, tissue homogenates were boiled in SDS sample buffer (3% SDS, 2 mM EDTA, 4% β-mercaptoethanol, 10% glycerol, 50 mM Tris, pH 6.8), followed by precipitation by 5 volumes of pre-chilled methanol at – 20 °C for 2 h, then centrifugation at 14,000 rpm for 30 min at 4 °C. The pellets were resuspended in 50 μL reducing buffer (8 M urea, 2% CHAPS, 5 mM tributylphosphine, 20 mM Tris, pH 8.0) for 1 h at room temperature (RT), then added 5 μL iodoacetimate (200 mM) in dark at RT for more than 1 h. Five volumes of pre-chilled methanol were added and incubated at – 20 °C for 2 h and centrifuged at 14,000 rpm for 30 min at 4 °C. The pellets were resuspended in 200 μL of rehydration buffer (7 M urea, 2 M thiourea, 1% DTT, 1% CHAPS, 1% Triton X-100, 1% ampholyte pH 3–10, trace of amount bromophenol blue) and centrifuged at 5000 rpm for 5 min at RT.

The samples were loaded onto the immobilized pH gradient strips for rehydration at RT for more than 12 h with gentle shaking. The first-dimensional isoelectric focusing was performed on the rehydrated gel strips for 7 h using a focusing tray. For the second dimension SDS-PAGE, the focused gel strips were equilibrated for 15 min each in equilibration buffer A (6 M urea, 2% SDS, 20% glycerol, 130 mM dithiothreitol, 0.375 M Tris–HCl, pH 8.8) and equilibration buffer B (6 M urea, 2% SDS, 20% glycerol, 135 mM iodoacetamide, 0.375 M Tris–HCl, pH 8.8), respectively. The equilibrated strips were loaded onto 15% Bio-Rad Criterion gels at 150 V for 90 min. The rest steps were the same as described below.

### Western Blotting

To detect the PK-resistant PrP (PrP^res^), the samples were incubated with PK at 100 μg/mL at 37 °C for an hour, shaking at 450 rpm, followed by addition of protease inhibitor mixture cocktail (Roche), and boiling at SDS sample buffer for 10 min in order to terminate the PK reaction, while samples without PK treatment were added to the sample buffer directly to detect untreated PrP. Samples were loaded onto 15% Tris–HCl Criterion pre-cast gels (Bio-Rad) for SDS-PAGE. After SDS-PAGE, the proteins on the gels were transferred to Immobilon-P polyvinylidene difluoride (PVDF, Millipore) for 100 min at 0.35 A. After blocking in TBS-Tween-20 buffer at RT for an hour, the membranes were incubated at RT with anti-PrP antibody 3F4 at 1:40,000 dilution overnight. The membranes were washed with washing buffer (1 × TBS, 0.1% Tween-20) for 5 min for 4 times, then incubated with horseradish peroxidase-conjugated sheep anti-mouse IgG at 1:3000 dilution at RT for 1 h, followed by washing 5 min for 4 times. The protein bands were visualized on Kodak film by ECL Plus following the product instruction.

### Statistical Analysis

To quantify the protein level, Western blots were scanned with an Epson Expression 1680 scanner (Epson America, Inc, Los Alamitos, CA). Protein intensity on the Western blots was quantified by densitometric analysis with less exposed films to avoid protein signal saturation and subtracting the background of films using UN-SCAN-IT gel Analysis Software (Silk Scientific, Inc., Orem, UT). The statistical differences in intensity of PrP detected by Western blotting or ThT fluorescence of PrP aggregates among different groups were statistically analyzed using Student’s *t*-test or one-way ANOVA to obtain *p* values. All tests adopted a two-sided type I error level of 0.05.

## Results

### TC-5RW-Treated Mice Have Much Longer Survival Time as well as Less PrP^Sc^ Deposition and Spongiform Degeneration Than Vehicle-Treated Mice

**Fig. 1:**
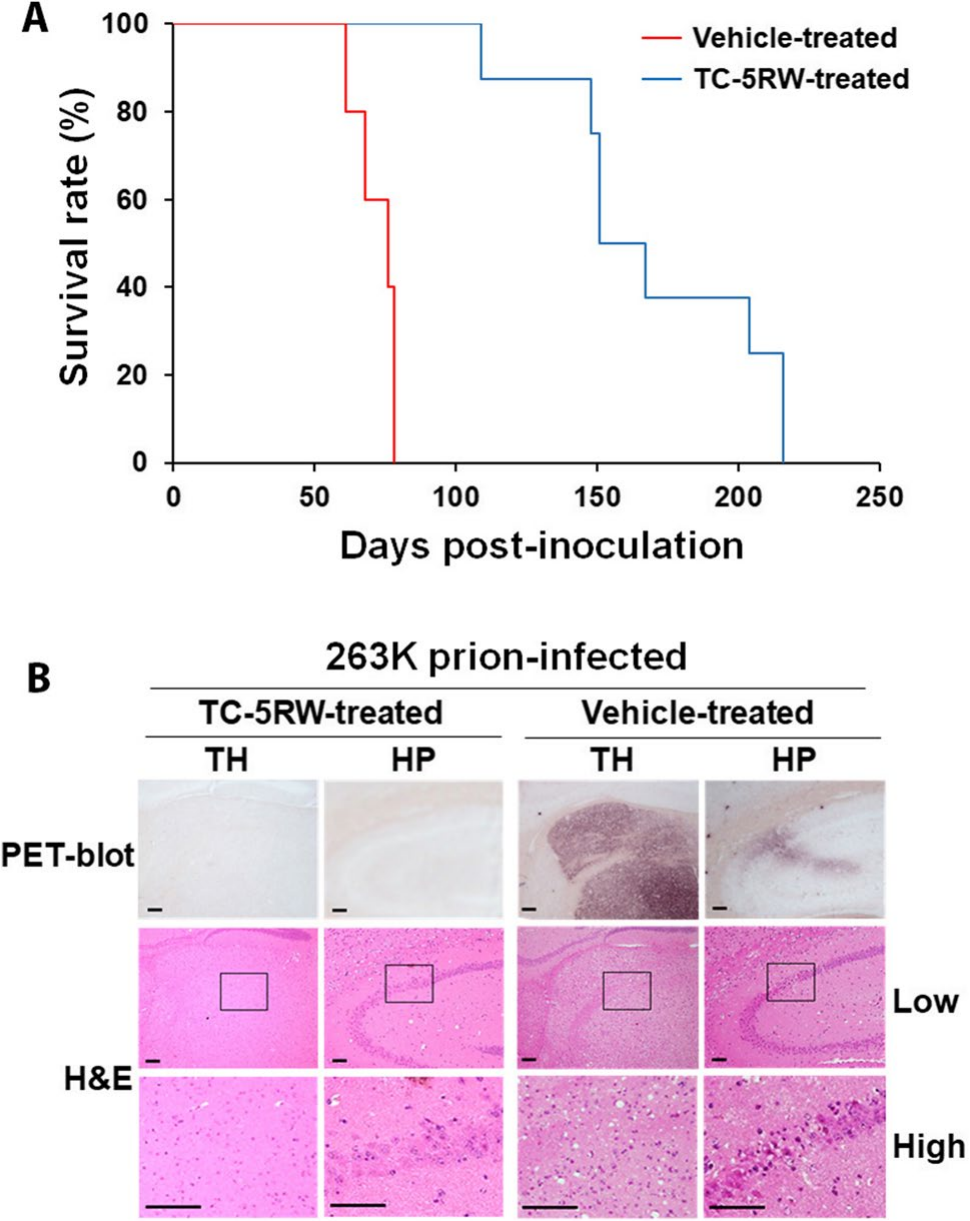
Therapeutic effect of TC-5RW. **A** Survival rate of 263 K-infected Tg7 mice given intraperitoneally either with a single TC-5WR dose (blue line) or with an equal volume of saline (vehicle controls, red line) on the same day for intracerebral inoculation with 263 K prion strain. **B** Histopathological examination of brain tissues of 263 K-infected Tg7 mice. Magnified PET-blot images and corresponding H&E staining images, obtained from the serial brain sections, are shown for the thalamus (TH) and hippocampus (HP) of the 263 K-infected mouse intraperitoneally given TC-5RW (sample #TC4) or saline (vehicle control; sample #V4). The areas surrounded by squares in the low-magnification photos are enlarged in the high-magnification images. The bar scale indicates 50 μm. It was noted that histopathological changes such as vacuolation in the thalamus and neuronal cell loss in the CA2 of the hippocampus were parallel to PrP^Sc^ deposition and much more remarkable in the vehicle control

Cellulose ethers including TC-5RW are able to extend the survival time of prion-infected rodents [8, 10]. To determine whether a potential therapeutic effect could be reflected in the prion-seeding activity of skin tissues of infected animals treated with the compound, we intracerebrally inoculated hemizygous Tg7 mice with the 263 K prions. On the same day as inoculation, infected Tg mice were injected intraperitoneally with a single dose of TC-5RW at 50 mg (*n* = 8) or with saline vehicle (*n* = 5). The survival days post-inoculation of the 263 K-infected Tg mice expressing hamster PrP were significantly longer in the drug-treated group than the vehicle-treated mice when a single intraperitoneal injection was administered on the same day of intracerebral infection (170 (mean) ± 38 (SD) vs 72 ± 8, days post-inoculation, *p* < 0.0005) (Fig. 1A). As previously reported by Doh-ura and co-workers, the compounds used in various combinations of timings and routes all exhibited a protective role in the infected animals [8]. To provide the proof-of-evidence, we selected one of the combinations for the compound administration in our current study. Our result indicated that TC-5RW effectively slowed the progression of the disease.

To determine how TC-5RW treatment affected PrP deposition and neuropathological changes in the brain of infected animals, we compared the PrP^Sc^ deposition and spongiform degeneration in the brain of animals treated with TC-5RW or saline vehicle using PET blotting and H&E staining. Compared to vehicle-treated mice (Fig. 1B, right upper panels), the TC-5RW-treated mice exhibited substantially decreased PrP^Sc^ staining in both the thalamus (TH) and the hippocampus (HP) brain regions (Fig. 1B, left upper panels). Remarkably, the spongiform degeneration was considerably decreased in the brain of TC-5RW-treated than vehicle-treated mice (Fig. 1B, lower panels). In sum, the TC-5RW treatment greatly prolonged the survival time and decreased PrP^Sc^ deposition and neuronal lesions in the brain of prion-infected mice.

### Prion-Seeding Activity Is Undetectable in the Skin Tissues of 263 K-Infected Mice Treated with TC-5RW by RT-QuIC and sPMCA

**Fig. 2:**
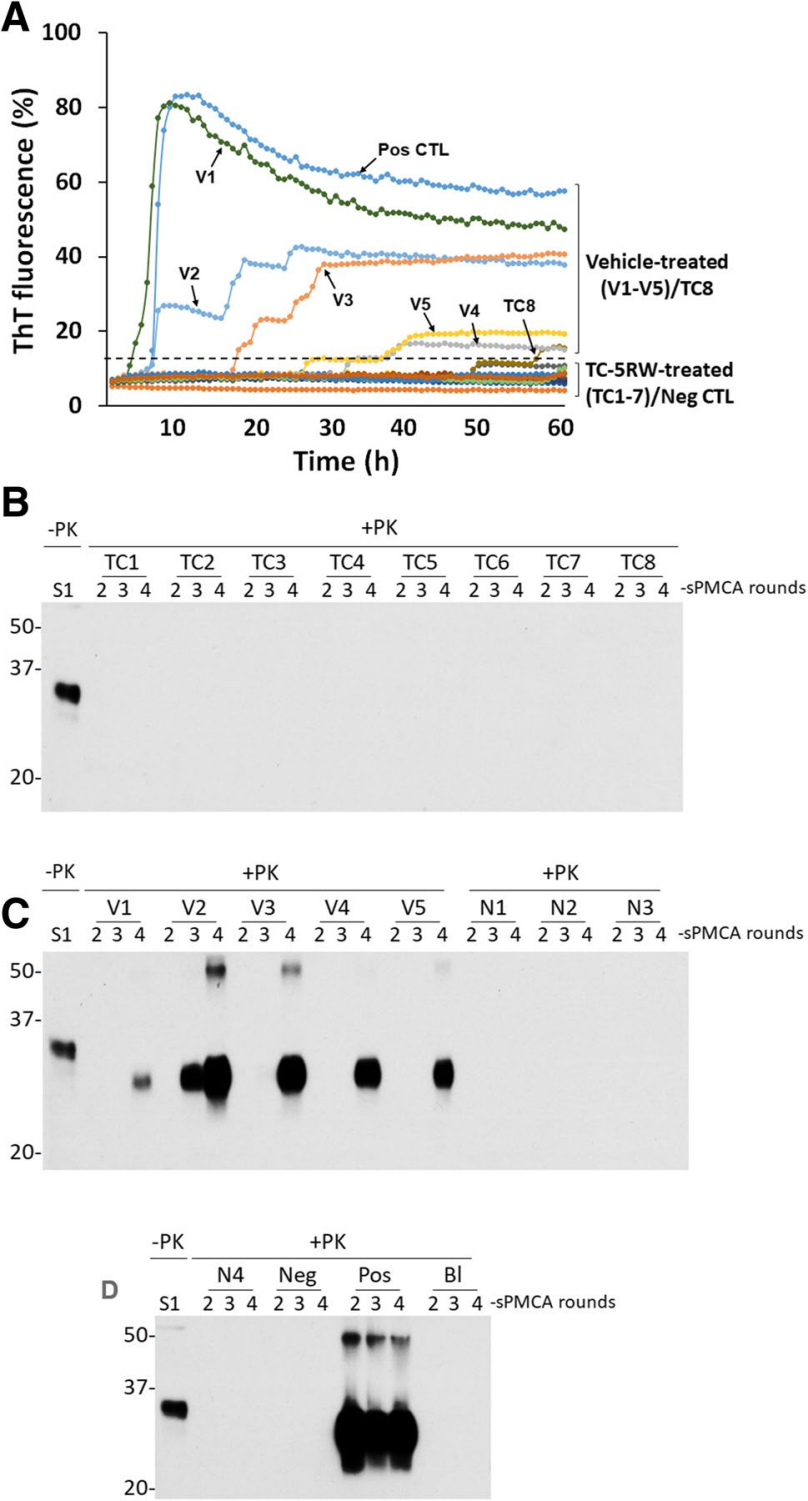
Detection of skin prion-seeding activity in 263 K-infected Tg mice by RT-QuIC and sPMCA. **A** Blinded RT-QuIC assay of prion-seeding activity in skin samples from 263 K-infected Tg7 mice treated with TC-5RW (TC1-8) or saline (vehicle-treated, V1-5). Pos CTL: positive controls with brain homogenate from a 263 K-infected hamster as the seed; Neg CTL: negative controls with brain homogenate from a healthy hamster as the seed. Dotted line represents the threshold that defines the positive and negative seeding activity based on the average ThT fluorescence of negative controls plus three SD. **B** Western blotting of blinded sPMCA products of skin samples from 263 K-infected Tg7 mice injected with TC-5RW (TC1-8). Normal brain homogenate without PK treatment was used as a control (S1). **C** Western blotting of blinded sPMCA products of skin samples from 263 K-infected Tg7 mice given saline (vehicle controls, V1-5) and non-infected Tg7 mouse controls (N1-3). **D** Western blotting of blinded sPMCA products of skin samples from non-infected Tg7 mouse (N4) as well as with normal control (Neg) and 263 K-infected hamster control (Pos) brain homogenate as seeds and no seeds control (blank: Bl). In the sPMCA experiment, there were 2 sets of positive and negative controls. The infected Tg mice treated with saline (vehicle controls, V1-V5) served as positive controls while uninfected Tg mice (N1-N4) served as negative controls for our animal study. In addition, the 263 K-infected hamster brain homogenates were used as positive controls while both uninfected hamster brain homogenates and sPMCA reaction without seeds (BI) served as negative controls of sPMCA to make sure that sPMCA worked. The sPMCA samples were 2–4 rounds of sPMCA products and were treated with PK prior to Western blotting probing with 3F4

To determine whether the extended incubation periods associated with TC-5RW-treatment correlate with any changes in the levels of prion-seeding activity in the skin, next we performed highly sensitive RT-QuIC and sPMCA assays for detection of skin prion-seeding activity. The RT-QuIC result demonstrated that similar to the negative control (Neg CTL) all TC-5RW-treated mice (TC1-TC7) exhibited virtually no skin ThT reaction except for one (sample TC8) that showed a very weak ThT reaction after 55 h; in contrast, skin samples of all prion-infected control mice treated with vehicle (V1-V5) were found positive ThT reaction, similar to the infected positive control (Pos CTL) (Fig. 2A). Moreover, consistent with RT-QuIC assay, 2–4 rounds of sPMCA detected no PrP^res^ in the skin tissues of the TC-5RW-treated mice (TC1-TC8), but PrP^res^ was detected in the skin from the vehicle-treated mice (V1-V5) (Fig. 2B–D). As controls, skin samples of infected Tg mice amplified PrP^res^ while skin samples from non-infected (N1-N4), negative control (Neg), and samples without seeds (Bl) showed no PrP^res^. Taken together, our RT-QuIC and sPMCA results indicate that TC-5RW treatment reduced the deposition and formation of PrP^Sc^ in the skin of infected Tg mice, reminiscent of its effect on brain PrP^Sc^.

### TC-5RW Inhibits Skin Prion-Seeding Activity and PrP^Sc^ Amplification In Vitro

**Fig. 3:**
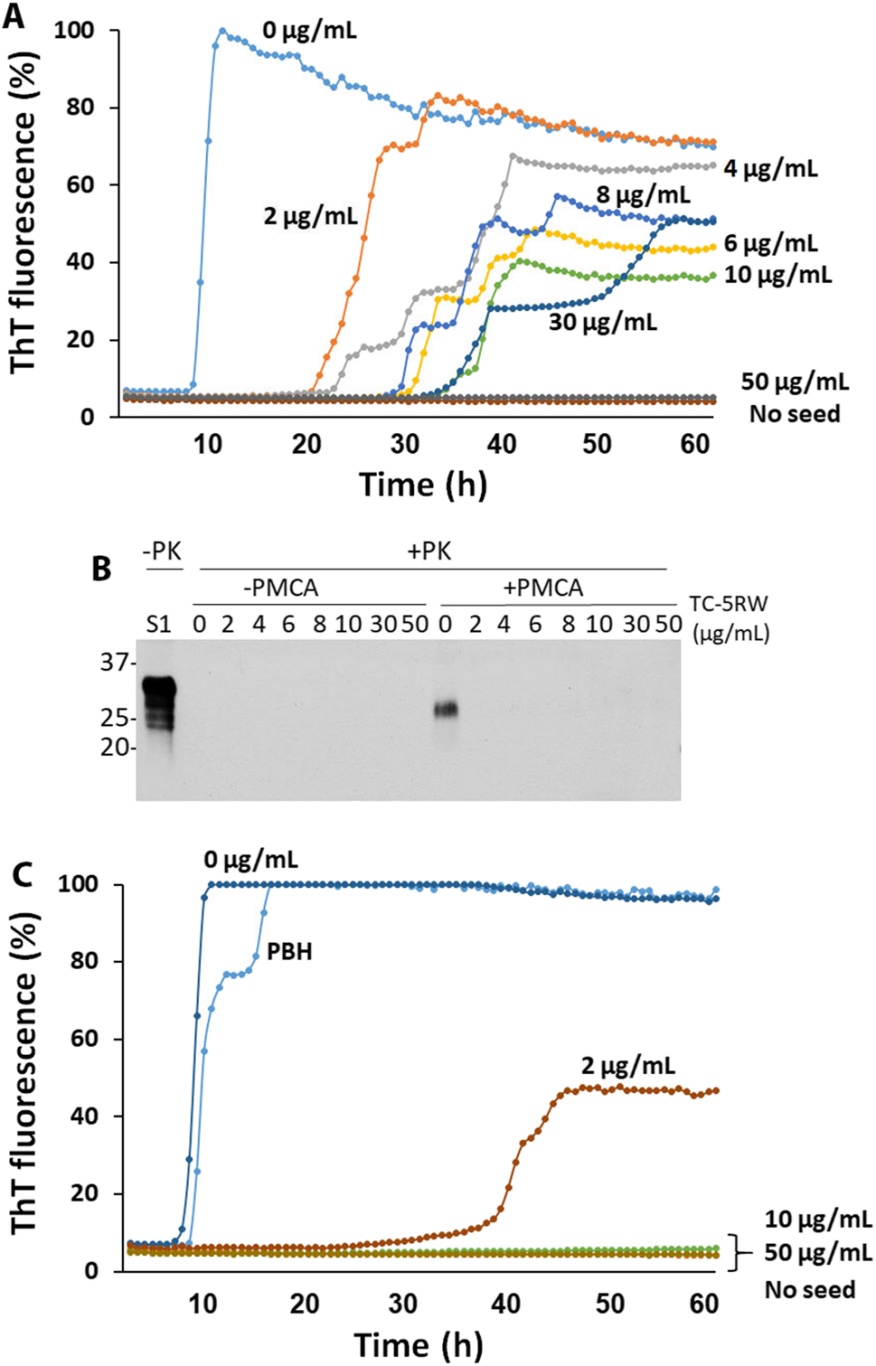
Inhibition of PrP^Sc^-seeding activity by TC-5RW in vitro. **A** RT-QuIC assay of prion-seeding activity of brain homogenates from 263 K-infected hamsters in the presence of different amounts of TC-5RW ranging from 0–50 μg/mL. **B** Western blotting of PK-resistant PrP^Sc^ of 263 K-infected hamster sPMCA products (+ PMCA, right panel) and samples without sPMCA (− PMCA, left panel) in the presence of different amounts of TC-5RW (0–50 μg/mL). **C** RT-QuIC assay of prion-seeding activity of skin homogenates from 263 K-infected Tg7 mice expressing hamster PrP^C^ in the presence of different amounts of TC-5RW ranging from 0–50 μg/mL. PBH, positive brain homogenate. No seed in **A** and **C** RT-QuIC without brain homogenate seeds as a control to exclude spontaneous seeding activity

Next, we determined whether the lack of detectable PrP^Sc^-seeding activity in skin tissues of animals treated with TC-5RW is due to the inhibition of the PrP^Sc^ amplification by residual TC-5RW left in the skin. Based on the previous observation, approximately less than 0.5–1 mg of TC-5RW/gram tissue was expected to be left in the skin sample [8]. To test this possibility, we added different amounts of TC-5RW ranging from 0 to 50 μg/mL into the RT-QuIC in vitro assay of brain homogenates from 263 K-infected animals. Dose-dependent inhibitory effect of TC-5RW on ThT fluorescence intensity was observed (Fig. 3A).

We also examined the effect of TC-5RW on sPMCA of brain PrP^Sc^ in vitro. We added different amounts of TC-5RW in the sPMCA substrates, with 263 K-infected hamster brain homogenate as seeds, to verify the influence of TC-5RW on sPMCA of brain PrP^Sc^. Compared to non-PMCA samples, amplification of PrP^Sc^ was only observed in the samples without TC-5RW but not in samples with different amounts of TC-5RW (Fig. 3B).

The same inhibitory effect of TC-5RW on prion-seeding activity was also detected by RT-QuIC assay in the skin samples. More than 50% ThT intensity of skin prion was inhibited in the presence of 2 μg/mL of TC-5RW while it was completely inhibited in the presence of 10 μg/mL or higher of TC-5RW (Fig. 3C). Moreover, the lag time of RT-QuIC was significantly increased compared to the sample without the compound (~ 38 vs ~ 8 h) (Fig. 3C).

### TC-5RW Treatment Does Not Affect 2D Profile of Brain PrP But Inhibits PrP^Sc^ Amplification by sPMCA In Vitro

**Fig. 4:**
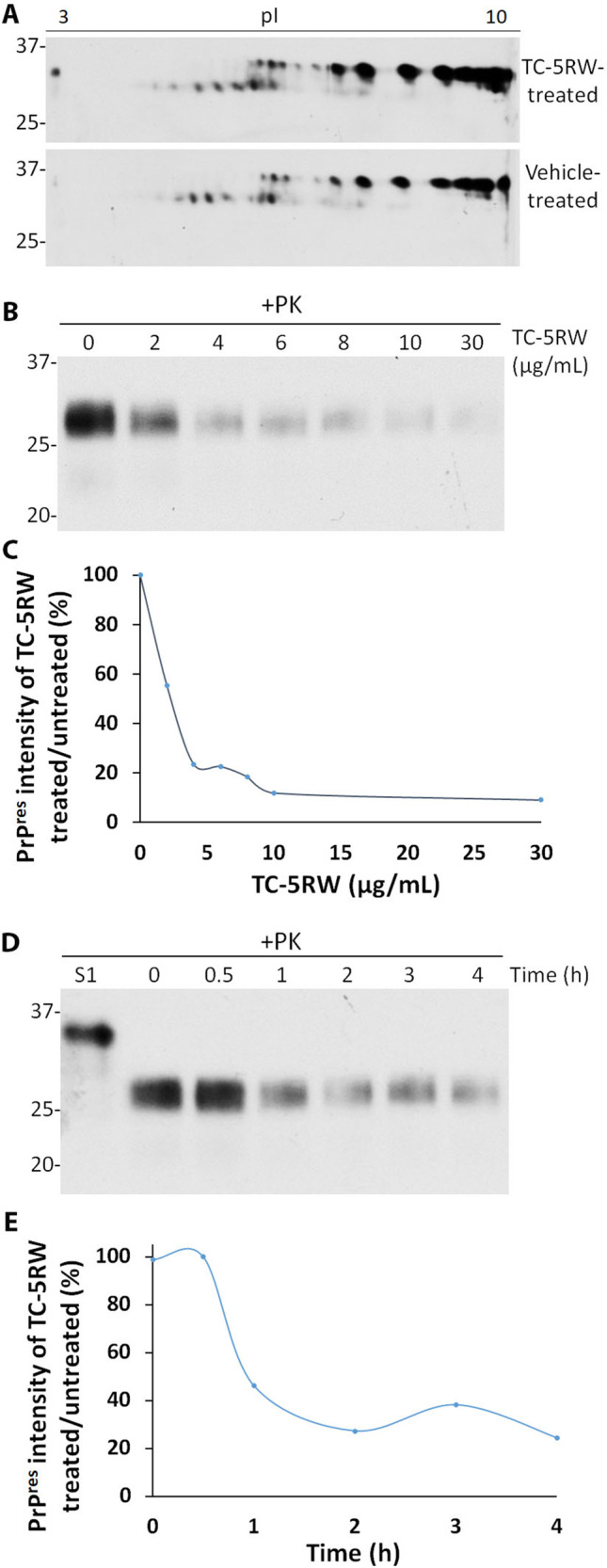
The effect of TC-5RW on hamster brain PrP in vivo and in vitro. **A** Two-dimensional (2D) analysis of brain PrP from 263 K prion-infected Tg7 mice injected with TC-5RW (TC-5RW treated) or saline (vehicle treated). **B** Representative Western blotting of PK-resistant PrP^Sc^ in the brain homogenates from 263 K-infected hamsters after incubation with different amounts of TC-5RW ranging from 0–30 μg/mL at 37 °C for 2 h. **C** Quantitative analysis of PrP^Sc^ percentage of TC-5RW-treated/untreated brain homogenates based on Western blot analysis in **B**. **D** Representative Western blotting of PK-resistant PrP^Sc^ in the brain homogenates from 263 K-infected hamsters after incubation with 10 μg/mL at 37 °C at different time points ranging from 0–4 h. **E** Quantitative analysis of PrP^Sc^ percentage of TC-5RW-treated/untreated brain homogenates based on Western blot analysis in **D**. S1: the supernatant of brain homogenate after low-speed centrifugation without PK treatment as a control

Two-dimensional (2D) gel electrophoresis coupled with Western blotting is a high-resolution technique that is able to reflect not only molecular weights but also charges of proteins interested, molecular characteristics that can be affected by therapeutic compounds. To determine whether TC-5RW treatment changes molecular weight and charges of PrP in the brain of prion-infected mice, we compared the 2D gel profile of PrP molecules from mice administered with TC-5RW or saline vehicle. 2D gel electrophoresis and Western blotting showed no differences in 2D gel profiles between the two groups (Fig. 4A), suggesting that the compound affects no post-translational modification. Most of the diglycosylated PrP spots were located on the basic side with pI 6–10 and molecular weights at 33–35 kDa, while most of the mono-glycosylated PrP spots migrated in the acidic side pI 4–6.5 and molecular weights at 27–29 kDa (Fig. 4A).

Consistent with previous findings [8, 10], our RT-QuIC and sPMCA assays also revealed that TC-5RW inhibited seeding activity and amplification of PrP^Sc^ from both brain and skin tissues in vitro (Figs. 2 and 3). However, it is unknown whether the compound has any direct effect on PrP^Sc^. To address this issue, we incubated brain homogenates of 263 K prion-infected hamster with different amounts of TC-5RW at 37 °C for 2 h. The levels of PrP^res^ were determined by western blotting after PK treatment of hamster 263 K brain homogenates that were subjected to incubation with different amounts of TC-5RW. We observed that the incubation of brain homogenates with TC-5RW at as low as 4 μg/mL decreased the intensity of PrP^res^ by approximately 80% when compared to untreated samples (Fig. 4B and 4C).

We also determined the effect of incubation time of TC-5RW with brain homogenates on the levels of PrP^res^. After incubation for 1 h, the levels of PrP^res^ were decreased approximately 60% when compared to that at the time zero (Fig. 4D and 4E). In sum, incubation of TC-5RW with PrP^Sc^ can directly reduce the level of hamster PK-resistant PrP^Sc^.

### TC-5RW Treatment Directly Decreases Human PK-Resistant PrP^Sc^ In Vitro

To date, the inhibitory effect of CEs on PrP^Sc^ conversion has been observed in rodent and other animal prions, but no studies with human prions have been reported. Next, we determined whether TC-5RW has an inhibitory effect on human PrP^Sc^. Different amounts of TC-5RW from 0–50 μg/mL were added into the sPMCA reaction in which PrP^Sc^ in brain homogenates from sCJDMM1 (MM1) or sCJDMM2 (MM2) were seeded in the normal brain homogenates of humanized Tg mice (Tg40h) expressing human wild-type PrP^C^ with 129-MM polymorphism [22]. The PK-resistant PrP^res^ was detected in the both sCJDMM1 and sCJDMM2 sPMCA products without TC-5RW. In contrast, no PrP^res^ was detected in the sPMCA products of sCJDMM1 or sCJDMM2 in the presence of TC-5RW (Supplementary Fig. 1). Although PrP^res^ was still detected in SCJDMM2 sPMCA product in the presence of 2 μg/mL of TC-5RW, its level was significantly decreased compared to that in the sample without TC-5RW.

**Fig. 5:**
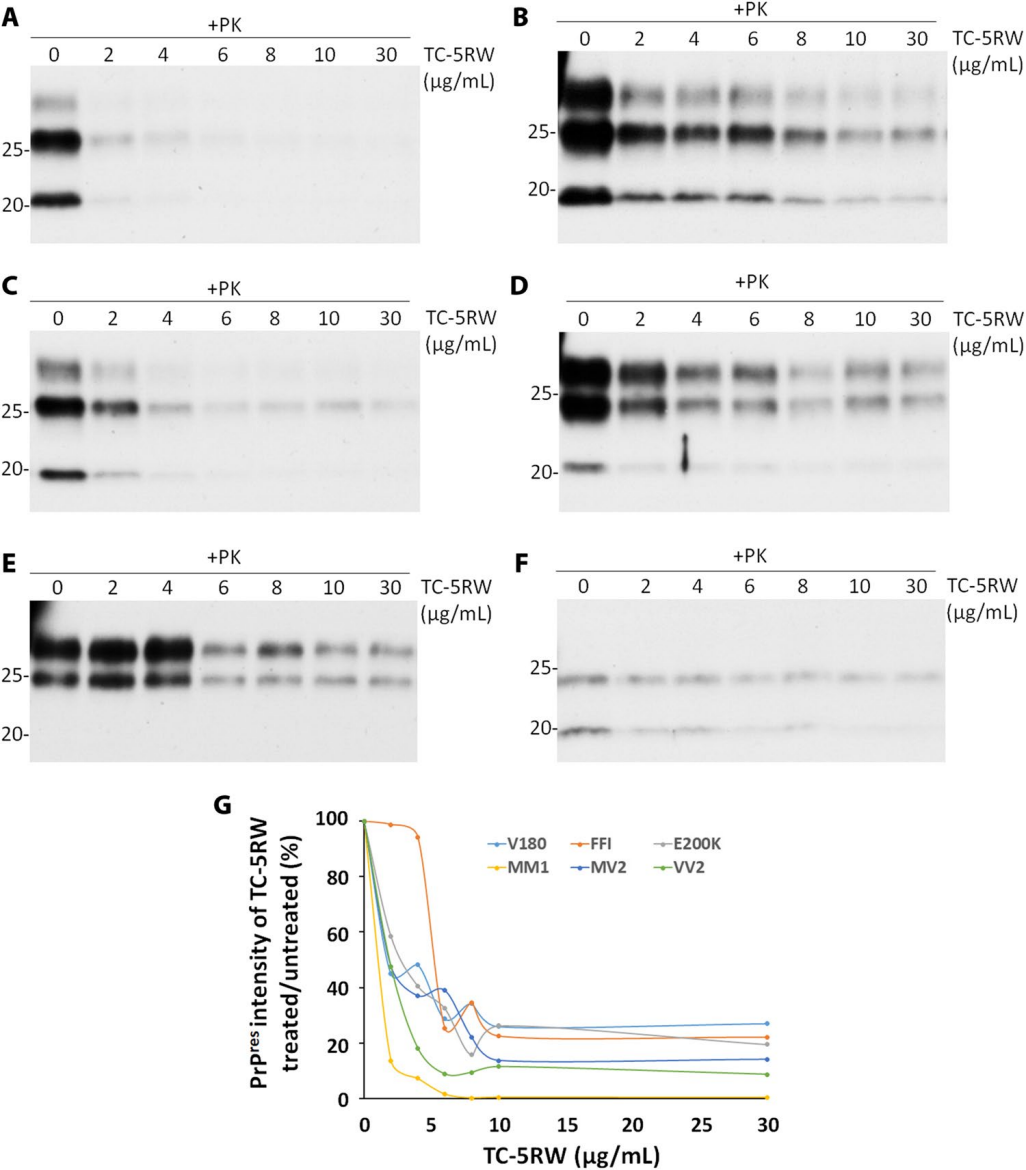
The effect of TC-5RW on human PK-resistant PrP^Sc^ by in vitro incubation. Representative Western blotting of human PK-resistant PrP^Sc^ in the brain homogenates from sCJDMM1 (MM1, **A**), sCJDMV2 (MV2, **B**), sCJDVV2 (VV2, **C**), fCJD^E200K^ (E200K, **D**), FFI (**E**), and fCJD^V180I^ (V180, **F**) after incubation with different amounts of TC-5RW ranging from 0–30 μg/mL at 37 °C for 2 h. **G** Quantitative analysis of PrP^Sc^ percentage of TC-5RW treated/untreated based on Western blot analyses in **A** through **F** above

To determine whether TC-5RW also has a direct effect on PK-resistant human PrP^Sc^, the brain homogenates from sCJDMM1, sCJDMV2, sCJDVV2, fCJD^E200K^, FFI, and fCJD^V180I^ were incubated with different amounts of TC-5RW (0–30 μg/mL) at 37 °C for 2 h. The level of PK-resistant PrP^Sc^ was detected by Western blotting probed with the anti-PrP antibody 3F4 after PK treatment. Similar to hamster 263 K prion, in vitro TC-5RW incubation significantly decreased the levels of PK-resistant PrP^Sc^ from various human prion diseases including most common subtypes of sCJD and fCJD at concentrations as low as 2 μg/mL, except for FFI that started to show a significant decrease at 6 μg/mL (Fig. 5). In addition, we also examined the effect of TC-5RW on human PrP^res^ at − 20 °C. Interestingly, after incubation of brain homogenates from sCJDVV2 with different amounts of TC-5RW from 0, 2, 4, 6, 8, 10, and 30 μg/mL, the levels of PrP^res^ were also dramatically decreased (Supplementary Fig. 2).

### TC-5RW Inhibits Seeding Activity of Prions from Various Human Prion Diseases In Vitro

**Fig. 6:**
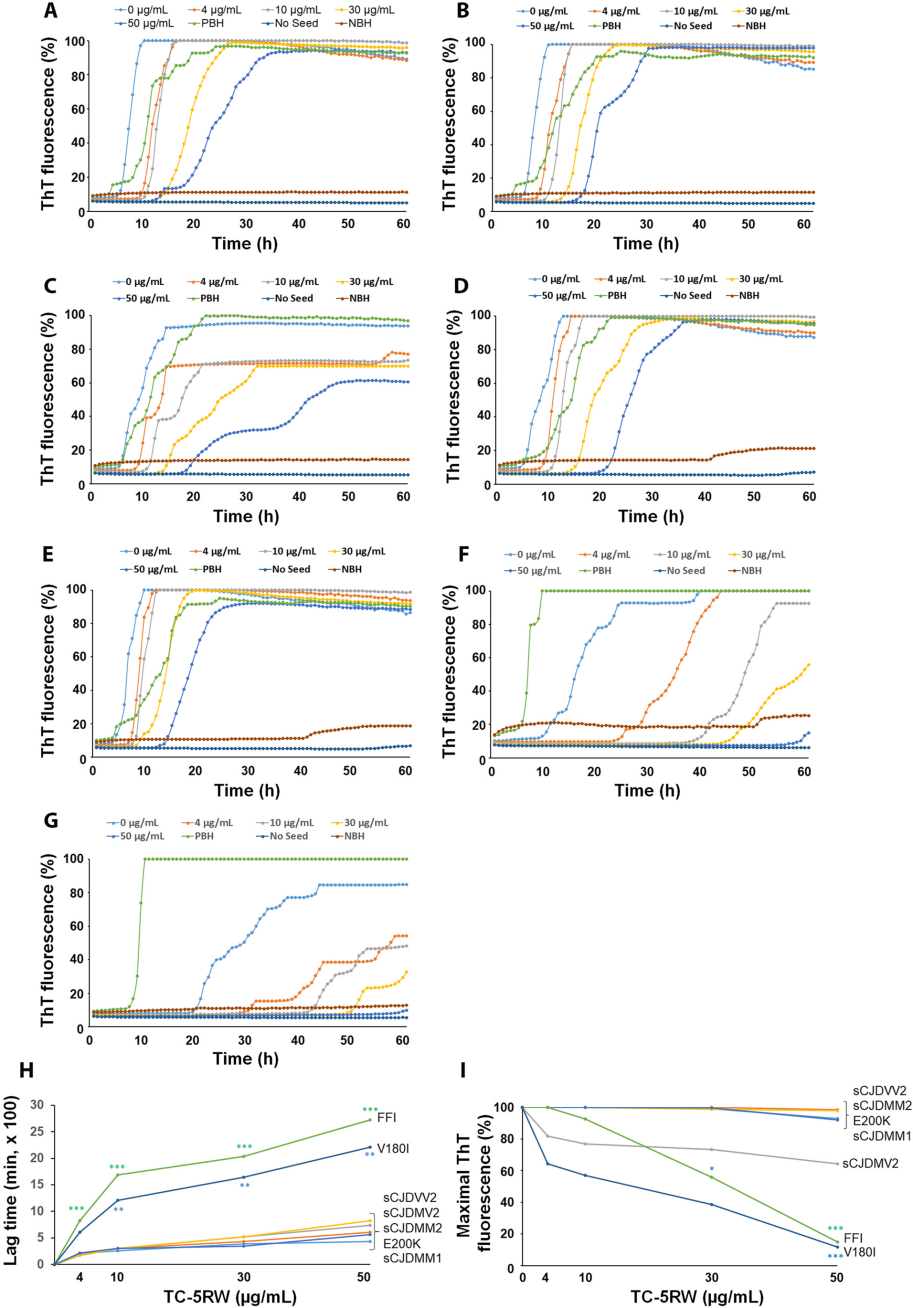
The effect of TC-5RW on seeding activity of PrP^Sc^ from various human prion diseases. RT-QuIC spectra of seeding activity of PrP^Sc^ in the brain homogenates from sCJDMM1 (**A**), sCJDMM2 (**B**), sCJDMV2 (**C**), sCJDVV2 (**D**), fCJD^E200K^ (**E**), FFI (**F**), and fCJD^V180I^ (**G**) in the presence of different amounts of TC-5RW ranging from 0–50 μg/mL. **H** Comparison of the lag time of seeding activity of PrP^Sc^ from sCJDMM1, sCJDMM2, sCJDMV2, sCJDVV2, fCJD^E200K^, FFI, and fCJD^V180I^. Green ***p < 0.001 for comparing lag time between FFI and sCJDMM1 at different TC-5RW concentrations. Blue **p < 0.01 for comparing the lag time between V180I and sCJDMM1 at different TC-5RW concentrations. **I** Comparison of ThT intensity at the endpoint of RT-QuIC reaction of PrP^Sc^ from sCJDMM1, sCJDMM2, sCJDMV2, sCJDVV2, fCJD^E200K^, FFI, and fCJD^V180I^. Green * and ***p < 0.05 or < 0.001 for comparing ThT fluorescence intensity between FFI and sCJDMM1 at TC-5RW concentration of 30 μg/mL or 50 μg/mL, respectively. Blue ***p < 0.001 for comparing ThT fluorescence intensity between V180I and sCJDMM1 at TC-5RW concentration of 50 μg/mL

We further investigated the effect of TC-5RW on the seeding activity of PrP^Sc^ from various human prion diseases including sCJDMM1, sCJDMM2, sCJDMV2, sCJDVV2, fCJD^E200K^, FFI, and fCJD^V180I^ by RT-QuIC assay (Fig. 6A through G). Although TC-5RW exhibited an inhibitory effect on prion-seeding activity in all human prion diseases, FFI and fCJD^V180I^ had better inhibitory effects compared to various subtypes of sCJD and fCJD^E200K^ in terms of the lag times (Supplementary Table 1) (Fig. 6H) and maximal intensity of ThT fluorescence at the endpoint of reaction (Supplementary Table 2) (Fig. 6I). Taken together, as done with hamster 263 K-prion, TC-5RW is also able to inhibit prion-seeding activity in various sporadic and genetic CJDs in vitro.

### TC-5RW Inhibits Seeding Activity of Prions from CWD Deer In Vitro

Consistent with the previous study reported by Hannaoui et al. [10], we also found that TC-5RW was able to inhibit the seeding activity of PrP^Sc^ from CWD deer in the RT-QuIC assay. The compound significantly prolonged the lag time of ThT reaction upon an increase in the concentrations of TC-5RW (Supplementary Fig. 3). The ThT fluorescence intensity was significantly decreased at 30 μg/mL while no ThT reaction was detected at 50 μg/mL of TC-5RW (Supplementary Fig. 3).

## Discussion

In addition to confirming the therapeutic effect of TC-5RW, a CE compound, on 263 K prion-infected animals, our current study made several important new findings. First, seeding activity and amplification of skin PrP^Sc^ were significantly inhibited in 263 K-infected animals treated with TC-5RW, reflecting the reduced levels of PrP^Sc^ in the brain after TC-5RW treatment. This may indicate prion seeding in the skin as a potential biomarker for monitoring the therapeutic efficacy of compounds. Second, TC-5RW exhibited an inhibitory effect on the seeding activity of PrP^Sc^ from various human prion diseases including sporadic and genetic CJD as well as FFI, suggesting that it may have therapeutic potential for human prion diseases. We also confirmed its inhibitory effect on CWD prions. Finally, incubation of PrP^Sc^ with TC-5RW directly decreased the level of PK-resistant PrP^Sc^, a function similar to detergents such as guanidine hydrochloride that may dissociate PrP^Sc^ aggregates, suggesting that it may be used for decontamination of prions.

There are no cures for the fatal transmissible prion diseases including CJD in humans. The lack of an operational assay to assess the therapeutic efficacy in clinical trials of prion diseases limits screening effective compounds for the treatment. The pathogenesis and disease progression of prion disease are highly associated with the accumulation of PrP^Sc^ in the brain. The current detection of PrP^Sc^ mainly depends on examination of the brain tissues or maybe the cerebrospinal fluid (CSF) for prion-seeding activity by RT-QuIC assay. However, due to the high risks of complications by these highly invasive procedures, it may not be practical to use brain biopsy or lumbar puncture for routine follow-up in clinical trials. Remarkably, our recent study indicated that PrP^Sc^ can be detected in the skin tissues of CJD patients [3] and could be a biomarker for early preclinical diagnosis of prion disease [5]. Our present study indicated that seeding activity and amplification capability of skin PrP^Sc^ were undetectable when the Tg7 mice were given therapeutic compound TC-5RW and showed prolonged lifespan compared to vehicle-treated mice. This finding provided a proof-of-concept evidence that skin prion-seeding activity may serve as a biomarker for monitoring therapeutic efficacy in clinical trials of prion diseases. Biomarkers have been believed as critical to the discovery and development of disease therapeutics. For instance, skin prion-seeding activity could provide an early indication of therapeutic target brain prion improvement, thereby adjusting clinical trial design and allowing successful therapeutic development. Moreover, skin prion-seeding activity may also allow evaluating therapeutic intervention on disease progression. A skin punch biopsy is a less invasive procedure than a spinal tap; it can be conducted for outpatients. Therefore, detection of prions in the skin would be a highly valuable biomarker for evaluation of therapeutic efficacy in clinical trials, in addition to the diagnosis of prion diseases. This can be tested in human prion-infected humanized mice in the future.

Cellulose ethers (CEs) have already widely been used as inactive ingredients in foods and pharmaceuticals. It has been observed that CEs do not modify prion protein expression but inhibit PrP^Sc^ formation in vitro and in prion-infected cells [8, 10]. Importantly, they have pre- or post-infection prophylactic effects and post-symptomatic therapeutic effects in prion-infected rodents [8]. Our current study observed that the CE compound TC-5RW is able to not only inhibit the seeding activity of PrP^Sc^ but also directly decrease PK-resistant PrP^Sc^ in various human prion diseases including the most common form of sCJD (sCJDMM1) and genetic CJD. Therefore, it is most likely that CEs can be used for clinical trials of human prion diseases. Especially, it will be of high clinical value for asymptomatic PrP mutation carriers since CEs have been proved to have pre-infection prophylactic effects in animals in the previous study [8]. Interestingly, we observed that TC-5RW showed a better inhibitory effect for two genetic prion diseases compared to other sCJD subtypes. However, this finding has not been validated in humans yet. Therefore, it will be interesting to determine whether CEs have a prophylactic effect in asymptomatic carriers of PrP mutations that are associated with various familial prion diseases. It has been known that most of these asymptomatic PrP mutation carriers will inevitably develop familial prion diseases during aging. It is possible that the incapability of RT-QuIC to detect skin prion-seeding activity in TC-5RW-treated Tg mice may partially result from the inhibitive effect of TC-5RW on seeding activity of RT-QuIC assay in vitro. However, our RT-QuIC assay revealed that the skin prion-seeding activity of RT-QuIC could not be completely inhibited until 50 μg/mL (Fig. 3A).

The exact mechanisms underlying extending survival time of infected animals by CEs remain unclear. Indeed, the levels of PK-resistant PrP^Sc^ have been found to decrease compared to the infected animals without CE treatment [10], consistent with our current finding with PET blotting of brain tissue sections from infected animals. Moreover, TC-5RW has been observed to inhibit the prion amplification capability of sPMCA and seeding activity of PrP^Sc^ seeds through RT-QuIC in vitro by previous studies [8, 10] and our current study. In addition, the inhibitory effect of CEs on PrP^Sc^ formation also was found in a cell-based model of prion disease [8, 11]. However, the CE concentration required for the inhibitory effect was believed to be dependent on the approach used [8]. For instance, the CE concentration of less than 10 μg/g tissue equivalent was needed for inhibition of hamster PrP^Sc^ in vivo and in vitro while it required ~ 1 mg/mL in the cell model [8]. Indeed, the decreased levels of PK-resistant PrP^Sc^ in the brain of CWD-infected Tg mice were proposed to result from the alteration in the PK resistance of PrP^Sc^ in the CE-treated mice [10]. However, it cannot be ruled out that the decrease in the levels of PK-resistant PrP^Sc^ in the brain of the CEs-treated mice could just result from the inhibitory effect of CEs on the conversion of PrP^C^ into PrP^Sc^ or PrP^Sc^ formation in vivo. This is because that the levels of PK-resistant PrP^Sc^ will be decreased if the PrP^Sc^ formation is inhibited in the brain of Tg mice treated by CE compounds. Our new finding that TC-5RW is able to directly decrease the PK-resistant PrP^Sc^ by direct incubation of brain homogenates with TC-5RW may represent another mechanism involved in its therapeutic effect. However, our study by in vitro directly incubating TC-5RW with brain homogenates from 263 K-infected hamsters, CWD prion-infected deer, and various human prions-infected humans provided direct evidence that TC-5RW indeed alters PK resistance of PrP^Sc^ either at 37 °C or even at − 20 °C. The decrease in the levels of PK-resistant PrP^Sc^ by the direct incubation of RT-5RW with brain homogenates is reminiscent of a phenomenon that has been well demonstrated with guanidine hydrochloride [25–27]. It is possible that as guanidine hydrochloride, TC-5RW is also able to dissociate PrP^Sc^ aggregates or change the conformation of PrP^Sc^. It has been shown that CEs induced the conformation transition of silk fibroin from random coil form to β-sheet structure [28]. In contrast, in the case of the effect of CEs on PrP^Sc^ conformation, whether TC-5RW may induce conversion of β-sheet structure into α-helix structure remains to be determined.

Notably, the TC-5RW-treated mice with longer survival time had very milder cerebral lesions in the hippocampus and thalamus compared to the vehicle-treated mice. However, they were clinically in the terminal stage of the disease and died of prion disease ultimately. This apparent discrepancy may result from a possibility that TC-5RW may inhibit disease progression less effectively in the brainstem than in the cerebrum. This possibility will be clarified in the future. The other possibility is that TC-5RW may just reduce PK-resistant PrP^Sc^ but not PK-sensitive PrP^Sc^, which may be echoed by the newly identified variably protease-sensitive prionopathy. The latter is characterized by the deposition in the brain of less PK-resistant PrP^Sc^ but more PK-sensitive PrP^Sc^ and exhibits a less severe brain damage and longer disease duration compared to sCJD [29–31].

## Supplementary Information

Below is the link to the electronic supplementary material.

Supplementary file1 (DOCX 224 KB)

## Data Availability

All materials used in this study will be made available subject to a material transfer agreement.

## Declarations

The use of autopsy human brain tissues was authorized by the Institutional Review Board of University Hospital Cleveland Medical Center and Case Western Reserve University, Cleveland, Ohio. The consents were received for each case through the National Prion Disease Pathology Surveillance Center (NPDPSC), Case Western Reserve University, Cleveland, Ohio, for research on retained tissues after written informed consents given by the patients during life or their next of kin after death. Postmortem examinations, if permission was available, were carried out in NPDPSC. All information was analyzed anonymously.

## Abbreviations

PrP^C^: Cellular prion protein
PrP^Sc^: Scrapie isoform of prion protein
sCJD: Sporadic Creutzfeldt-Jakob disease
RT-QuIC: Real-time quaking-induced conversion
PMCA: Protein misfolding cyclic amplification
Tg: Transgenic
2D: Two-dimensional electrophoresis
