# Supplementary material for: Decrease in Skin Prion-Seeding Activity of Prion-Infected Mice Treated with a Compound Against Human and Animal Prions: a First Possible Biomarker for Prion Therapeutics
Source: Mol Neurobiol. 2021 May 13;58(9):4280–92. doi: 10.1007/s12035-021-02418-6 (PMC8487418; doi:10.1007/s12035-021-02418-6)

Decrease in skin prion-seeding activity of prion-infected mice treated with a compound against human and animal prions: a first possible biomarker for prion therapeutics

Mingxuan Ding^1,2^, Kenta Teruya^3^, Weiguanliu Zhang^1,2^, Hae Weon Lee^2^, Jue Yuan^2^, Ayumi Oguma^3^, Aaron Foutz^2^, Manuel V. Camacho^2^, Marcus Mitchell^2^, Justin J. Greenlee^4^, Qingzhong Kong^2,5^, Katsumi Doh-ura^3§^, Li Cui^1§^, Wen-Quan Zou^2,5§^

^1^Department of Neurology, The First Hospital of Jilin University, Changchun, Jilin Province, China.

^2^Departments of Pathology and Neurology, Case Western Reserve University School of Medicine, Cleveland, Ohio 44106, USA.

^3^Department of Neurochemistry, Tohoku University Graduate School of Medicine, 2-1 Seiryo-machi, Aoba-ku, Sendai, Miyagi 980-8575, Japan.

^4^Virus and Prion Research Unit, National Animal Disease Center, USDA, Agricultural Research Service, 1920 Dayton Avenue, Ames, IA 50010, USA.

^5^National Prion Disease Pathology Surveillance Center, Case Western Reserve University School of Medicine, Cleveland, Ohio 44106, USA.

§Corresponding authors: Wen-Quan Zou: [wxz6@case.edu](mailto:wxz6@case.edu); Li Cui: [chuili1967@126.com](mailto:chuili1967@126.com); Katsumi Doh-ura: [doh-ura@med.tohoku.ac.jp](mailto:doh-ura@med.tohoku.ac.jp)

**Supplementary Tables**

Table 1

| **Table 1 Effect of TC-5RW on lag time of prion seeding activity among prion diseases** | | | | | |
| --- | --- | --- | --- | --- | --- |
| **Diseases** | **Ave (SD) or comparison** | **P values of lag time comparison at different amounts of TC-5RW (min)** | | | |
|  |  | **4 µg/ml** | **10 µg/ml** | **30 µg/ml** | **50 µg/ml** |
| **MM1** | Ave (SD) | 245 (25.1) | 288.7 (25.7) | 490.3 (90.5) | 591.3 (195.4) |
|  | P, vs MM2 | 0.057 | 0.105 | 0.236 | 0.585 |
|  | P, vs MV2 | 0.994 | 0.494 | 0.391 | 0.157 |
|  | P, vs VV2 | 0.069 | 0.76 | 0.475 | 0.202 |
|  | P, vs E200K | 0.055 | 0.103 | 0.125 | 0.611 |
|  | P, vs FFI | 0.0002*** | 6.83E-06*** | 0.0004*** | 7.09E-06*** |
|  | P, vs V180I | 0.094 | 0.007** | 0.0016** | 0.0033** |
| **MM2** | Ave (SD) | 159 (50.2) | 245.7 (24.8) | 389.7 (86.5) | 519.7 (75.1) |
|  | P, vs MV2 | 0.368 | 0.296 | 0.145 | 0.102 |
|  | P, vs VV2 | 0.728 | 0.273 | 0.092 | 0.017* |
|  | P, vs E200K | 0.994 | 0.348 | 0.513 | 0.997 |
|  | P, vs FFI | 0.0002*** | 5.971E-06*** | 0.0003*** | 3.471E-06*** |
|  | P, vs V180I | 0.073 | 0.006** | 0.0012** | 0.0017** |
| **MV2** | Ave (SD) | 245.7 (139.2) | 360.7 (163.9) | 606.3 (188.6) | 1111.3 (479.1) |
|  | P, vs VV2 | 0.439 | 0.613 | 0.656 | 0.303 |
|  | P, vs E200K | 0.365 | 0.196 | 0.095 | 0.106 |
|  | P, vs FFI | 0.0019** | 0.0002*** | 0.001** | 0.006** |
|  | P, vs V180I | 0.1 | 0.0113* | 0.004** | 0.062 |
| **VV2** | Ave (SD) | 173.3 (43.5) | 303.7 (75.1) | 548.3 (89.1) | 779.3 (86.5) |
|  | P, vs E200K | 0.72 | 0.154 | 0.057 | 0.035* |
|  | P, vs FFI | 0.0002*** | 2.27E-05*** | 0.0004*** | 8.23E-06*** |
|  | P, vs V180I | 0.076 | 0.008** | 0.0019** | 0.0037** |
| **E200K** | Ave (SD) | 158.7 (49.7) | 202.3 (66.2) | 332 (109.3) | 519.3 (114.3) |
|  | P, vs FFI | 0.0002*** | 1.36E-06*** | 0.0003*** | 1.05E-05*** |
|  | P, vs V180I | 0.072 | 0.0055** | 0.0011** | 0.002** |
| **FFI** | Ave (SD) | 938 (90.1) | 1688.3 (75.1) | 2077.3 (229) | 2611 (66.1) |
|  | P, vs V180I | 0.849 | 0.342 | 0.12 | 0.031* |
| **V180I** | Ave (SD) | 1010 (606.3) | 1442.3 (388) | 1686 (257) | 1976.3 (330.5) |
| Note: *: *p* < 0.05; ** *p* < 0.01; *** *p* < 0.001. | | |  |  |  |

Table 2

| **Table 2 Comparison of effect of TC-5RW on maximal ThT fluorescence of prion seeding activity among different prion diseases** | | | | | |
| --- | --- | --- | --- | --- | --- |
| **Diseases** | **Ave (SD) or comparison** | **P values of lag time comparison at different amounts of TC-5RW (min)** | | | |
|  |  | **4 µg/ml** | **10 µg/ml** | **30 µg/ml** | **50 µg/ml** |
| **MM1** | Ave (SD) | 1 (0) | 1 (0) | 0.995 (0.008) | 0.950 (0.032) |
|  | P, vs MM2 | / | / | 0.392 | 0.253 |
|  | P, vs MV2 | 0.374 | 0.374 | 0.36 | 0.283 |
|  | P, vs VV2 | / | / | 0.448 | 0.264 |
|  | P, vs E200K | / | / | 0.715 | 0.62 |
|  | P, vs FFI | / | 0.374 | 0.059 | 7.40E-05*** |
|  | P, vs V180I | 0.154 | 0.125 | 0.0293* | 6.88E-06*** |
| **MM2** | Ave (SD) | 1 (0) | 1 (0) | 0.9998 (0.0003) | 0.984 (0.028) |
|  | P, vs MV2 | 0.374 | 0.374 | 0.353 | 0.243 |
|  | P, vs VV2 | / | / | 0.377 | 0.861 |
|  | P, vs E200K | / | / | 0.151 | 0.287 |
|  | P, vs FFI | / | 0.374 | 0.0574 | 5.84E-05*** |
|  | P, vs V180I | 0.154 | 0.125 | 0.0286* | 4.59E-06*** |
| **MV2** | Ave (SD) | 0.804 (0.340) | 0.751 (0.431) | 0.711 (0.476) | 0.635 (0.440) |
|  | P, vs VV2 | 0.374 | 0.374 | 0.406 | 0.247 |
|  | P, vs E200K | 0.374 | 0.374 | 0.357 | 0.326 |
|  | P, vs FFI | 0.374 | 0.54 | 0.66 | 0.133 |
|  | P, vs V180I | 0.493 | 0.529 | 0.343 | 0.114 |
| **VV2** | Ave (SD) | 1 (0) | 1 (0) | 0.968 (0.056) | 0.980 (0.022) |
|  | P, vs E200K | / | / | 0.414 | 0.305 |
|  | P, vs FFI | / | 0.374 | 0.074 | 5.37E-05*** |
|  | P, vs V180I | 0.154 | 0.125 | 0.035* | 3.23E-06*** |
| **E200K** | Ave (SD) | 1 (0) | 1 (0) | 0.997 (0.003) | 0.924 (0.080) |
|  | P, vs FFI | / | 0.374 | 0.058 | 0.0003*** |
|  | P, vs V180I | 0.154 | 0.125 | 0.029* | 8.78E-05*** |
| **FFI** | Ave (SD) | 1 (0) | 0.925 (0.129) | 0.558 (0.289) | 0.150 (0.076) |
|  | P, vs V180I | 0.154 | 0.189 | 0.46 | 0.571 |
| **V180I** | Ave (SD) | 0.566 (0.428) | 0.506 (0.442) | 0.349 (0.337) | 0.120 (0.034) |
| Note: *: p < 0.05; ** p < 0.01; *** p < 0.001. | | |  |  |  |

**Supplementary Figure legends**

**Supplementary Figure 1. Western blotting of PrP^res^ after PMCA in the presence of different amounts of TC-5RW.** PMCA was conducted with the brain homogenates from normal humanized transgenic mice (Tg40h) expressing human wild-type PrP-129MM seeded with brain homogenates from cadavers with either sCJDMM1 or sCJDMM2 in the different amounts of TC-5WR from 0 to 50 µg/mL. The PrP in the PMCA products was treated with PK at 100 µg/mL, 37°C for 1 h prior to western blotting probed with 3F4.

**Supplementary Figure 2. Western blotting of PK-resistant PrP^res^ in brain homogenates from sCJDVV2 incubated with TC-5RW at -20°C.** Representative western blotting of PrP^res^ from brain homogenates of sCJDVV2 after incubated with different amounts of TC-5RW ranging from 0, 2, 4, 6, 8, 10, and 30 µg/mL at -20°C for 2 hours. Probed with 3F4.

**Supplementary Figure 3. RT-QuIC spectra of prion-seeding activity of brain homogenates of CWD deer.** Prion-seeding activity of brain homogenates of deer with CWD was examined in the presence of different amounts of TC-5RW (0-50 µg/mL). PBH: Positive brain homogenate from sCJD as a positive control. NBH: negative brain homogenate from non-CJD subject. No seeds: a RT-QuIC reaction without brain homogenate, which was used as another negative control to exclude possible spontaneous seeding activity.

**Supplementary Figures**

Supplementary figure 1


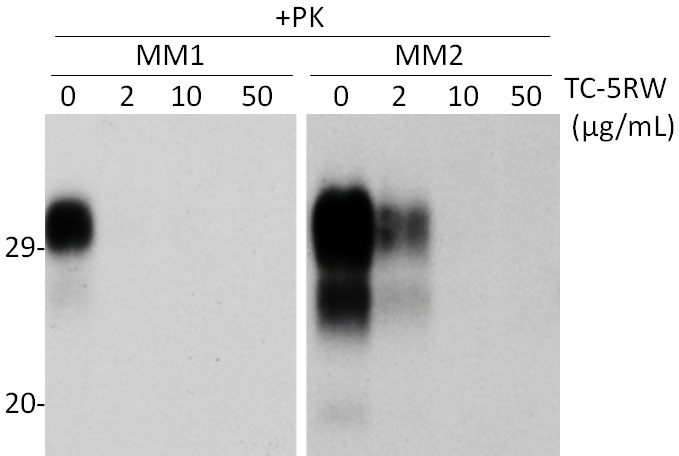


Supplementary Figure 2


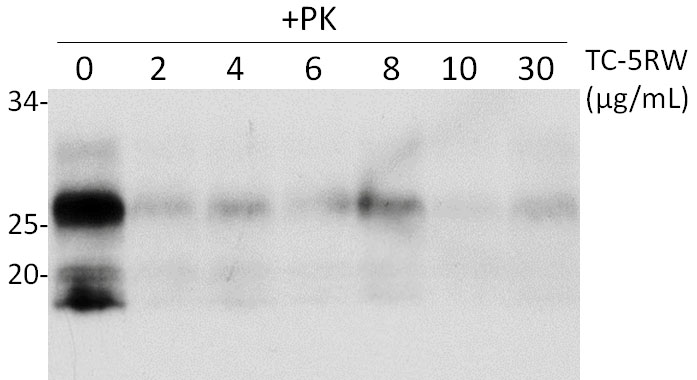


Supplementary Figure 3


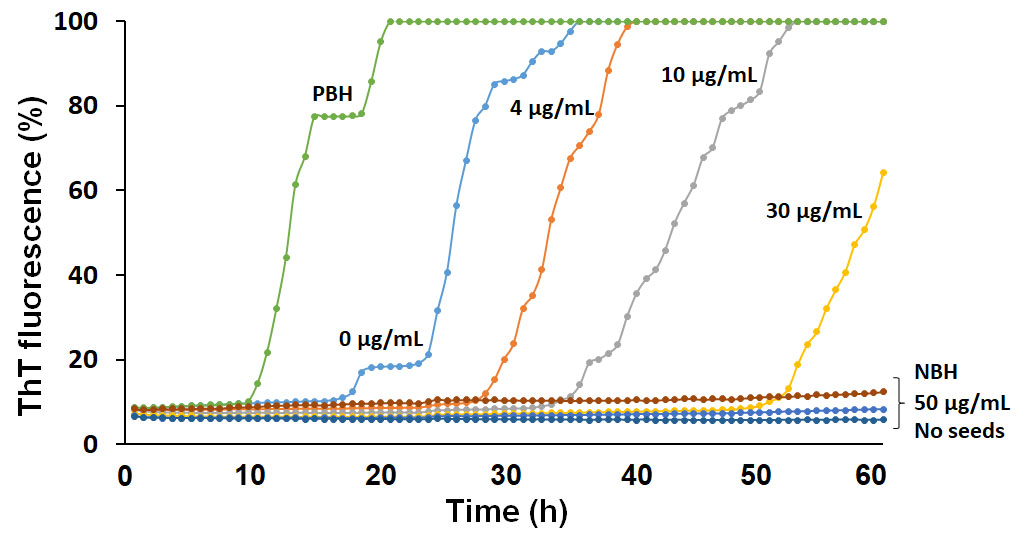

Supplement: Supplementary file 1 — Supplementary file1 (DOCX 224 KB) [file 12035_2021_2418_MOESM1_ESM.docx]
